# Complex patellofemoral reconstruction leads to improved physical and sexual activity in female patients suffering from chronic patellofemoral instability

**DOI:** 10.1007/s00167-020-06340-7

**Published:** 2020-10-29

**Authors:** Patricia M. Lutz, Philipp W. Winkler, Marco-Christopher Rupp, Stephanie Geyer, Andreas B. Imhoff, Matthias J. Feucht

**Affiliations:** 1 Department for Orthopedic Sports Medicine, Technical University Munich, Ismaninger Str. 22, 81675 Munich, Germany; 2 Department of Orthopedics and Trauma Surgery, Medical Center, Faculty of Medicine, Albert-Ludwigs-University of Freiburg, Freiburg, Germany

**Keywords:** Patellofemoral instability, Quality of Life, Return to sports, MPFL, Sexual activity, Sex

## Abstract

**Purpose:**

To analyze postoperative physical and sexual activity as well as Quality of Life (QoL) after complex patellofemoral reconstructions in female patients suffering from chronic patellofemoral instability (PFI).

**Methods:**

Female patients aged > 18 years undergoing complex patellofemoral reconstruction for chronic PFI were included. Complex patellofemoral reconstruction was defined as medial patellofemoral ligament reconstruction (MPFL-R) combined with at least one major bony procedure (distal femoral osteotomy, high tibial osteotomy, and trochleoplasty). Outcome was evaluated retrospectively after a minimum follow-up of 12 months using Tegner activity scale, Banff Patellofemoral Instability Instrument 2.0 (BPII 2.0), EuroQol-5D-3L (EQ-5D-3L), EuroQol Visual analog scale (EQ-VAS), and a questionnaire about sexual activity.

**Results:**

A total of 34 females (mean age, 26 ± 5 years) with a mean follow-up of 45 ± 16 months were included. Seventy-seven percent had one major bony correction + MPFL-R and 24% had at least two major bony corrections + MPFL-R. The re-dislocation rate was 6%. Median Tegner activity scale improved from 3 (range 0–10) to 4 (range 2–6) (n.s.) and an improved activity level was observed in 49% of subjects. QoL scores showed an EQ-5D-3L Index Value of 0.89 ± 0.15, EQ-VAS of 80.3 ± 11.4, and BPII of 68.3 ± 19.1. Thirty-four percent of patients reported restrictions of sexual activities due to PFI preoperatively with an improved sexual function observed in 60% postoperatively due to less pain, improved mobility, and less apprehension. Postoperative return to sexual activity was 91%, whereof 19% reported current restrictions of sexual function because of pain and/or limited range of motion.

**Conclusion:**

Despite the complexity and invasiveness of complex patellofemoral reconstruction, combined bony procedures and MPFL-R resulted in a low redislocation rate, improved physical activity and QoL comparable to values reported after isolated MPFL-R. Furthermore, sexual activity was improved in 60% of females with preoperative restrictions.

**Level of evidence:**

IV.

**Electronic supplementary material:**

The online version of this article (10.1007/s00167-020-06340-7) contains supplementary material, which is available to authorized users.

## Introduction

Patellofemoral instability (PFI) includes recurrent dislocation and subluxation of the patella with the highest prevalence observed in young females [11, 15]. PFI results in significant morbidity and functional impairment with a decreased physical activity level [6, 7, 11, 15, 34, 36, 43].

Given the complex interaction between dynamic muscle action, passive soft tissue restrains, surface geometry of the patellofemoral joint, and limb alignment, PFI is based on a multifactorial pathogenesis [10, 27, 46]. Anatomic risk factors, including trochlear dysplasia, valgus malalignment, torsional deformity, patella alta, and a lateralized position of the tibial tuberosity as well as insufficiency of the medial patellofemoral ligament (MPFL) must be taken into consideration for surgical treatment [3, 10, 27, 28, 47]. Whereas isolated MPFL reconstruction (MPFL-R) may be appropriate in most patients, complex reconstruction procedures are indicated in patients with additional risk factors [10]. Combined MPFL-R and bony corrections such as realignment osteotomies and trochleoplasty are invasive and technically demanding, but have proven to be effective for the treatment of chronic PFI [13, 24, 26, 38].

Over the last years, research on PFI has mainly focused on functional outcomes [1, 4, 13, 14, 24, 26, 35, 44], re-dislocation rates [10, 12, 15, 18, 43], and return to sports [30, 36, 45]. Recently, research concerning Quality of Life (QoL) after MPFL-R has gained more attention [4, 5, 17, 19, 21, 22]. Patient-reported QoL outcome measures are commonly seen as multi-dimensional and can therefore be influenced through a range of domains [23]. To date, however, only limited data are available about QoL after complex patellofemoral reconstruction procedures. Furthermore, chronic PFI may also lead to restrictions of sexual activity in young female patients. However, this aspect of QoL has not been investigated for patients with chronic PFI so far.

Better knowledge of postoperative outcomes concerning physical and sexual activity and QoL after complex patellofemoral reconstruction may help to justify such comprehensive procedures and may improve preoperative patient counseling regarding postoperative expectations.

The purpose of this study was to analyze physical activity, QoL, and sexual activity in female patients after complex patellofemoral reconstructions for chronic PFI. The hypothesis was that complex patellofemoral reconstruction improves physical and sexual activity as well as QoL.

## Materials and methods

This retrospective study was conducted to assess physical and sexual activity, as well as QoL after complex patellofemoral reconstructions in young female patients with chronic PFI. The study was approved by the institutional review board of the Technical University of Munich (575/19 S) and conducted according to the Declaration of Helsinki. All subjects gave their written informed consent to participate in this investigation.

For the purpose of this study, only female patients undergoing complex patellofemoral reconstruction (as defined below) between 07/2014 and 06/2019 for chronic PFI were included. Further inclusion criteria were: age > 18 years and postoperative follow-up of at least 12 months. Exclusion criteria were: relevant comorbidities (infectious diseases, cancer, severe cardiovascular diseases), relevant previous operations independent of operations on the patellofemoral joint, pregnancy at the time of data collection, lack of German language skills, and missing consent.

Complex patellofemoral reconstruction was defined as MPFL-R with or without tibial tubercle osteotomy (TTO) combined with at least one major bony procedure, including distal femoral osteotomy (DFO), high tibial osteotomy (HTO), and trochleoplasty. Indications for major bony procedures were: valgus malalignment > 5°, internal femoral torsion > 30°, external tibial torsion > 40°, and trochlear dysplasia Types B–D according to the Dejour classification [8]. TTO was indicated in patients with a tibial tubercle–trochlear groove (TT–TG) distance > 25 mm or patella alta.

### Data collection

Medical records were reviewed to collect patient demographics and details about the medical history and surgery.

Physical activity was evaluated by the Tegner activity scale [48] and questions regarding sports ability and frequency.

Global QoL was assessed using EuroQol-5D-3L (EQ-5D-3L) and EuroQol Visual analog scale (EQ-VAS) [16, 41, 42]. The EQ-5D-3L measures health related Quality of Life with regard to the 5 dimensions: mobility, self-care, usual activities, pain/discomfort, and anxiety/depression. Each dimension has 3 levels: no problems, some problems, and extreme problems. The descriptive system of the EQ-5D-3L produces a 5-digit health state profile which is then converted to an index value ranging from 0 (death) to 1 (full health). The EQ-VAS records the patient´s self-rated health on a visual analog scale with the endpoints “best imaginable health state” and “worst imaginable health state”.

Disease-specific QoL was assessed using the Banff Patellofemoral Instability Instrument 2.0 (BPII) [2, 20, 31]. In this score patients mark their answers on a visual analog scale. A higher score reflects a higher QoL [23].

Sexual activity was analyzed with a self-designed questionnaire inspired by Nunley et al*.* [40], which included questions about sexual restrictions pre- and postoperatively and changes in sexual activity postoperatively (Appendix 1).

### Statistical analysis

Statistical analysis was performed using SPSS software version 25.0 (IBM-SPSS, New York, USA). Continuous variables were calculated as mean ± standard deviation allowing one decimal (except for EQ-5D-3L Index Value, allowing two decimals). Categorical variables were reported as count and percentages allowing no decimal. Normal distribution of all data was evaluated with the Kolmogorov–Smirnov test. Longitudinal dependent samples were computed by the paired Wilcoxon signed-rank test for nonparametric data (Tegner activity scale) or with the Chi-square test (sexual restrictions and patellar dislocations pre-/postoperatively). Correlations were calculated using Pearson’s correlation coefficient for parametric data and Spearman’s correlation coefficient for nonparametric data allowing three decimals. A *p* value of less than 0.05 was considered to indicate statistical significance.

A retrospective power analysis revealed that with 34 patients, this study has a power of 0.8 to demonstrate a small to moderate effect of *d* = mean change/standard deviation of 0.5 in a *t*-test for paired samples. Similarly, to be able to obtain a correlation coefficient that significantly differs from *r* = 0 with a true correlation of 0.5, the power is 0.8 [49].

## Results

A total of 34 female patients were included for detailed analysis. Descriptive statistics of the demographical data and the main parameters are shown in Table 1. MPFL-R (± TTO) combined with one major bony procedure was performed in 77% of patients and combined MPFL-R (± TTO) with two or more major bony procedures was performed in 24% (Table 2). Preoperatively, 57% of the total study group suffered from patellar dislocations at least monthly. Postoperatively, one patient suffered from one patellar re-dislocation and another patient from at least two patellar re-dislocations (re-dislocation rate: 6%).

**Table 1:** Descriptive statistics of the demographical data and main parameters of the total study group

Variable	Total study group
Number of included patients	34
Follow-up [months]	45.3 ± 15.7 (12–72)
Age [years]	25.9 ± 5.2 (18–42)
BMI [kg/m^2^]	24.2 ± 6.0 (17.4–43.3)
In a relationship	
Yes	23 (68%)
No	11 (32%)
Patella dislocation before surgery	
Less than 1 per year	10 (29%)
Annually	5 (15%)
Monthly	8 (24%)
Weekly	5 (15%)
Daily	6 (18%)
Patella re-dislocation after surgery	
No re-dislocation	32 (94%)
One re-dislocation	1 (3%)
Two or more re-dislocations	1 (3%)

Continuous variables are shown as mean ± standard deviation (range), categorical variables are shown as number of patients and percentages of the total patient cohort

**Table 2:** Descriptive statistics of the surgical interventions of the total study group

Surgical interventions	Prevalence, *n* (%)
MPFL-R^§^ + DFO	18 (53%)
MPFL-R* + Trochleoplasty	7 (21%)
MPFL-R* + HTO	1 (3%)
MPFL-R* + DFO + Trochleoplasty	6 (18%)
MPFL-R + DFO + HTO + Trochleoplasty	1 (3%)
MPFL-R + HTO + Trochleoplasty	1 (3%)

Categorical variables are shown as number of patients and percentages of the total patient cohort

*DFO* distal femoral osteotomy, *MPFL-R* reconstruction of medial patellofemoral ligament with autologous gracilis tendon, *HTO* high tibial osteotomy, *TTO* tibial tubercle osteotomy

*Concomitant TTO was performed in 1 patient (3%)

^§^Concomitant TTO was performed in 2 patients (6%)

### Physical activity

Results of physical activity outcome scores are summarized in Table 3. The median Tegner score improved from 3 to 4, which was not statistically significant (*p* = 0.067). An improved sports ability was reported by 49% of patients whereas 18% reported a decreased sports ability. Postoperatively, the most commonly performed sport activities were cycling (70%), swimming (64%), fitness (55%), Nordic walking (30%), hiking (30%), and dancing (30%).

**Table 3:** Physical activity outcome scores of the total study group

	Total study group
TEGNER current	4* (range 2–6)
TEGNER preoperative	3* (range 0–10)
Change in sports ability postoperatively^§^	
No change, *n* (%)	11 (33%)
Improved, *n* (%)	16 (49%)
Deteriorated, *n* (%)	6 (18%)
Reasons for sports reduction postoperatively^$^	
Knee joint complaints, *n* (%)	6 (55%)
Complaints in other parts of body, *n* (%)	3 (27%)
Other reasons (family, career, other interests), *n* (%)	4 (36%)

*TEGNER* Tegner activity scale

*Values are median; categorical variables are shown as number of patients and percentages of the total patient cohort

^§^Question was answered by 33 patients

^$^Multiple answers possible; affects 11 patients who have indicated that the sport frequency has been reduced postoperatively

### Quality of Life

Mean EQ-VAS was 80.3 ± 11.4 (range 50–100) and mean EQ-5D-3L Index Value was 0.89 ± 0.15 (CI 0.84–0.94). Table 4 shows descriptive statistics of the five dimensions of QoL. Mean BPII 2.0 was 68.3 ± 19.1 (range 31–97). Significant correlations between results of EQ-VAS and BPII 2.0 (*r* = 0.725; *p* < 0.001) and EQ-5D-3L Index value and BPII 2.0 (*r* = 0.638; *p* < 0.001) could be determined.

**Table 4:** Descriptive statistics of the five dimensions of Quality of Life with Euro-QoL-Score (EQ-5D-3L)

Dimension	Level 1	Level 2	Level 3
Mobility	28 (82%)	6 (18%)	
Self-care	34 (100%)		
Usual activities	31 (91%)	3 (9%)	
Pain/discomfort	14 (41%)	19 (56%)	1 (3%)
Anxiety/depression	26 (77%)	7 (21%)	1 (3%)

Level 1: indicating no problem, Level 2: indicating some problems, Level 3: indicating extreme problems; categorical variables are shown as number of patients and percentages of the total patient cohort

### Sexual activity

Results of the questionnaire regarding sexual activity are summarized in Table 5. In total, 34% of patients reported restrictions of sexual activities due to knee problems preoperatively, whereas 19% reported restrictions postoperatively. The most common reasons for preoperative restrictions of sexual activity were pain followed by apprehension. Of patients with preoperative restrictions, 60% reported improvement postoperatively.

**Table 5:** Descriptive statistics of sexual activity

Variable	Total study group
Restriction of sexual activity due to knee joint complaints preoperatively^a^	
Yes, *n* (%)^a,^*	11 (34%)
Because of pain	8 (25%)
Because of limited mobility	6 (19%)
Because of apprehension	7 (22%)
No, *n* (%)	21 (66%)
Sexual activity postoperatively	
Yes, *n* (%)	29 (91%)
No, *n* (%)*	3 (9%)
Because of knee joint complaints	0
Because of other reasons	3 (9%)
Current restrictions of sexual activity due to knee joint complaints^b^	
Yes, *n* (%)*	6 (19%)
Because of pain	6 (19%)
Because of limited mobility	4 (13%)
No, *n* (%)	25 (81%)
Patellar dislocation during sexual activities preoperatively^a^	
Yes, *n* (%)	2 (6%)
No, *n* (%)	30 (94%)
Patellar dislocation during sexual activities postoperatively^c^	
Yes, *n* (%)	0 (0%)
No, *n* (%)	29 (100%)
Change of quality of sexual activities postoperatively^d^	
No change, *n* (%)	3 (30%)
Improved, *n* (%)^*^	6 (60%)
Because of less pain	4 (40%)
Because of improved mobility	3 (30%)
Because of less apprehension	5 (50%)
Deteriorated, *n* (%)	1 (10%)
Change of quantity of sexual activities postoperatively^d^	
No change, *n* (%)	7 (70%)
More frequently, *n* (%)^*^	3 (30%)
Because of less pain	3 (30%)
Because of improved mobility	2 (20%)
Because of less apprehension	2 (20%)
Less frequently, *n* (%)	0 (0%)

Categorical variables are shown as number of patients and percentages of patients

*Multiple answers possible

^a^*n* = 32

^b^*n* = 31

^c^*n* = 29

^d^Affects 11 patients who reported restrictions of sexual activities due to knee problems preoperatively, of those only 10 patients answered this question

## Discussion

This study has several important findings. First, in young female patients with chronic PFI and anatomic risk factors, complex patellofemoral reconstruction with combined MPFL-R and bony procedures can reliably restore patellar stability, with a relatively low re-dislocation rate of 6%. Second, despite the complexity and invasiveness of the procedures, sports ability could be improved in approximately 50% of patients. Third, QoL after complex patellofemoral reconstructions can be compared to QoL after isolated MPFL-R, as reported in the existing literature. No restrictions in self-care and only limited restrictions in usual activities and mobility could be found. Fourth, one third of female patients experienced restrictions in sexual activities due to problems associated with chronic PFI preoperatively. However, improved sexual activity was observed in 60% postoperatively.

The main intention of the present study was to describe the real-life benefit of comprehensive and invasive surgical procedures in this young patient cohort rather to describe score-based outcomes after a specific surgical procedure. Research after combined bony reconstructions and MPFL-R in patients with PFI concentrated on functional outcomes so far [1, 13, 24, 26, 38], and important measures such as QoL have been largely neglected. In recent years, it has generally been recognized that QoL is an important outcome parameter after orthopedic interventions. Therefore, the BPII was specifically designed for patients with chronic PFI. However, current research on QoL in patients with PFI concentrated on outcomes after isolated MPFL-R only [17–19, 21]. More recently, orthopedic studies have also focused on sexual activity outcomes [29, 33, 39, 40]. To the best of our knowledge, this aspect has not been investigated in patients with chronic PFI so far.

The present study focused on outcomes of female patients since young female patients have a higher risk of a primary and recurrent patellar dislocations [7, 11, 15] and tend to predispose to poorer functional outcomes [9, 25] as well as poorer QoL outcomes after MPFL-R [5, 17].

The re-dislocation rate has been considered a common outcome parameter of studies evaluating surgical treatment of chronic PFI. Comparable to our results, re-dislocation rate ranged from 0 to 5% in previous research after isolated MPFL-R [5, 9, 18, 21, 25, 44, 45]. Re-dislocation rate was 0% after derotational osteotomies of the distal femur and concomitant procedures (MPFL-R, patellofemoral arthroplasty, trochleoplasty, TTO) in patients with PFI during the follow-up period [26, 38] and comparably low after combined trochleoplasty and MPFL-R [1, 24].

Since PFI results in decreased physical activity levels, physical activity remains an important outcome parameter postoperatively. In a systematic review, Schneider et al. reported a mean Tegner activity score of 5.7 points after isolated MPFL-R [45]. Several other authors reported similar results after isolated MPFL-R [4, 25]. In a study of Howells et al., 76% of subjects had resumed sporting activities at a mean follow-up of 16 months [25]. Comparable to the results of Imhoff et al. and Nelitz et al. after DFO and MPFL-R in patients with chronic PFI, mean Tegner activity scale in our cohort was lower [26, 38]. It can, therefore, be concluded that activity levels after complex patellofemoral reconstructions remain limited compared to isolated MPFL-R. Nevertheless, nearly half of subjects in the present study claimed that physical activity improved postoperatively.

Another important finding of the present study was that QoL outcomes after complex reconstructions on the patellofemoral joint were comparable to the results after isolated MPFL-R or MPFL-R with concomitant TTO [17, 19, 21, 22]. Since the BPII is a patient-reported, disease-specific QoL score which is valid, reliable, and responsive in this patient population [20, 31], we conclude that one or more additional bony intervention does not deteriorate the QoL outcomes in patients with chronic PFI. It must be noted, however, that QoL includes multidimensional aspects and can be affected by the patient’s emotional and physical health, functional ability, and social environment [17]. Other studies assessed QoL after isolated MPFL-R with the EQ-5D-3L score [4, 5]. After a comparable follow-up period, Biesert et al. described a mean EQ-5D-3L VAS of 70 and an EQ-5D-3L Index of 0.78 in a Swedish cohort [4]. After a shorter follow-up period, Bouras et al. found a mean EQ-5D-3L VAS of 92 and an EQ-5D-3L Index of 1 in an English cohort [5]. Our results lie in between even though the surgical intervention was more extensive in our cohort. Compared to a representative survey in Germany [37], results of EQ-5D-3L VAS in our cohort were slightly below results of 25–44-year-old women in the mentioned study investigating health status in adults in Germany (80.3 versus 89). In the present study, psychological aspects, such as pain/discomfort and anxiety/depression seemed to substantially influence QoL postoperatively. This assumption is further strengthened by previous research [5, 44]. A non-significant improvement in the anxiety and depression scores postoperatively was reported by Bouras et al. [5]. Even though the BPII is a disease-specific QoL outcome measure and the EQ-5D-3L a standardized generic measure of health status that can be applied to a wide range of health conditions, significant correlations between all QoL outcome scores could be found in the present study.

To the best of our knowledge, this is the first study to describe sexual activity outcomes after complex reconstructive knee surgery for chronic PFI. Sexual dysfunction shows a strong association with physical and emotional dissatisfaction and depression [32]. We therefore believe that improved sexual activity is an important goal after a complex reconstruction on the patellofemoral joint, especially in a young patient cohort. The percentage of patients sexually active postoperatively in our cohort was more than 90%. This study demonstrated that most young female patients returned to their baseline or higher level of sexual activity after a complex surgical intervention because of PFI. Improvements in quality and frequency could be noted, caused by less pain, improved mobility, and less apprehension. In the life of a young women, difficulties during sexual activity can cause severe stress that could negatively influence QoL [33]. We, therefore, believe that it is important for an orthopedic surgeon to be able to make scientifically based statements on this subject in patient education, especially because young patients are affected.

This study has several limitations. First, this is a retrospective analysis without a control group. On the other hand, there are only few studies reporting on physical activity and QoL, and no study reporting on sexual activity after complex patellofemoral procedures. Second, the patient cohort is heterogenic concerning the surgical intervention, which is, however, attributable to the multifactorial pathogenesis of PFI. This can also be considered a strength, as the cohort is representative of the clinical situation. However, the main intention of the present study was not to describe the results of one specific procedure, but to describe physical activity, QoL, and sexual activity after invasive and complex patellofemoral reconstructions.

The results of this study have clinical relevance when considering postoperative outcomes, including physical and sexual activity and QoL, after complex patellofemoral reconstruction in patients suffering from chronic PFI. In the future, findings of this study can help to justify such comprehensive procedures and to improve preoperative patient counseling regarding postoperative expectations.

## Conclusion

Complex patellofemoral reconstruction, defined as combined major bony procedures and MPFL-R plus/minus TTO in female patients suffering from chronic PFI leads to improved physical activity. QoL outcomes are comparable to outcomes after isolated MPFL-R as reported in the existing literature. Furthermore, this is the first study to describe limitations in sexual activity in female patients with PFI and that restrictions of sexual function can be limited postoperatively.

## Electronic supplementary material

Below is the link to the electronic supplementary material.

Supplementary file1 (PNG 358 kb)
