# Supplementary figures and images for: Complex patellofemoral reconstruction leads to improved physical and sexual activity in female patients suffering from chronic patellofemoral instability
Source: Knee Surg Sports Traumatol Arthrosc. 2020 Oct 29;29(9):3017–24. doi: 10.1007/s00167-020-06340-7 (PMC8384801; doi:10.1007/s00167-020-06340-7)

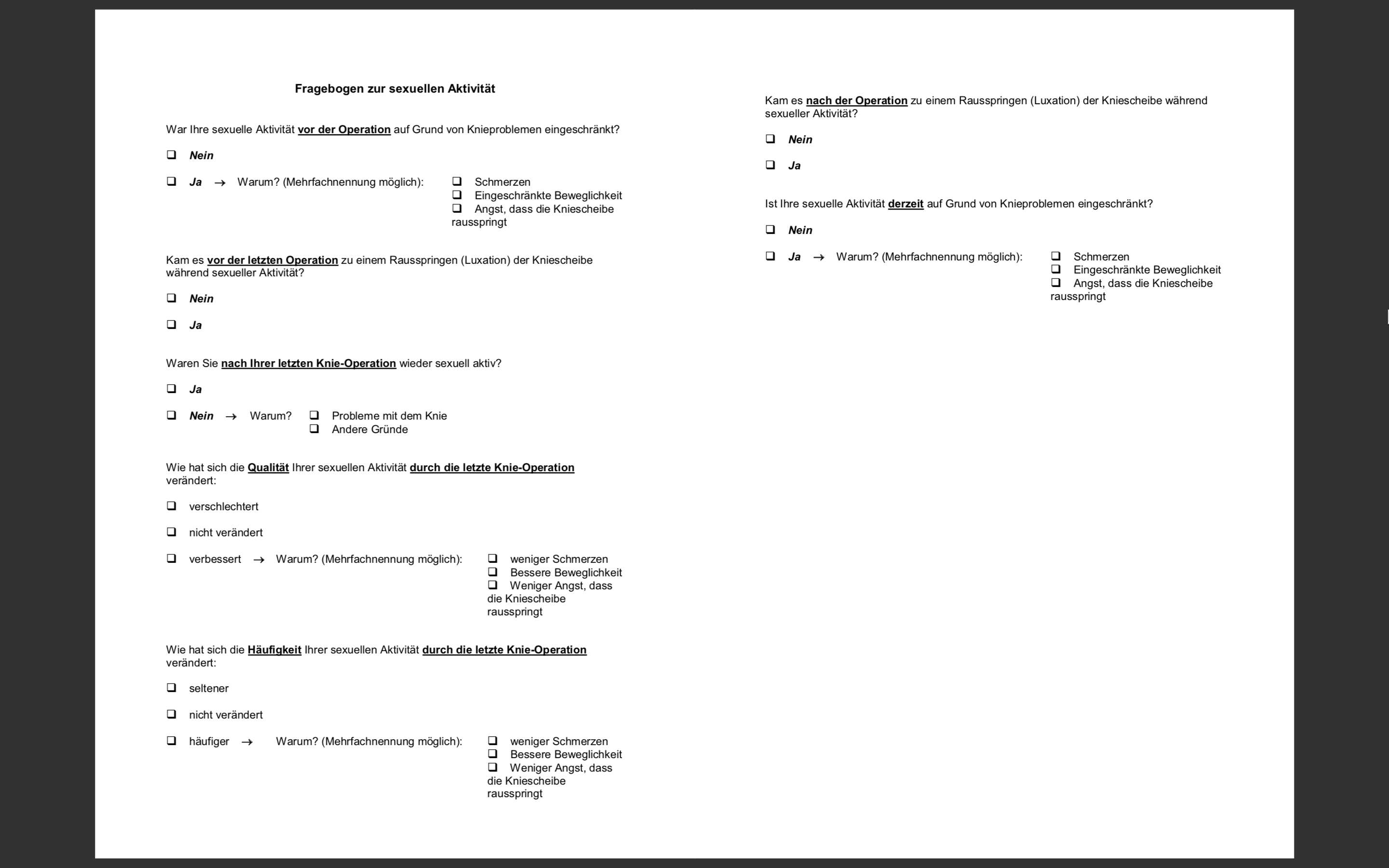

Supplement: Supplementary file 1 — Supplementary file1 (PNG 358 kb) [file 167_2020_6340_MOESM1_ESM.png]
